# Elevated Serum Level of HMGB1 in Patients with the Antiphospholipid Syndrome

**DOI:** 10.1155/2017/4570715

**Published:** 2017-12-19

**Authors:** Valeria Manganelli, Antonella Capozzi, Simona Truglia, Cristiano Alessandri, Emanuela Lococo, Tina Garofalo, Caterina De Carolis, Fabrizio Conti, Guido Valesini, Maurizio Sorice, Agostina Longo, Roberta Misasi

**Affiliations:** ^1^Department of Experimental Medicine, Sapienza University, Rome, Italy; ^2^Lupus Clinic, Dipartimento di Medicina Interna e Specialità Mediche, Sapienza University, Rome, Italy; ^3^Gynecology and Obstetrics, “San Giovanni Addolorata” Hospital, Rome, Italy

## Abstract

Pregnancy problems are common in patients with rheumatic disease; indeed, autoimmune disorders and autoantibodies can affect pregnancy progress and lead to maternal complications. Recent studies have highlighted a close association between HMGB1, chronic inflammation, and autoimmune diseases. Thus, in this investigation, we analyzed serum levels of HMGB1, an alarmin which plays a pivotal role in inducing and enhancing immune cell function. Sera from 30 patients with antiphospholipid syndrome (11 primary and 19 secondary APS), 35 subjects with pregnancy morbidity, and 30 healthy women were analysed for HMGB1 and its putative receptor RAGE (sRAGE) by Western blot and for TNF-*α* by ELISA. Results revealed that APS patients showed significantly increased serum levels of HMGB1, sRAGE, and the proinflammatory cytokine TNF-*α*, as compared to healthy women. However, also, the pregnancy morbidity subjects showed significantly increased levels of HMGB1 and sRAGE as well as TNF-*α* compared to healthy women. Our findings suggest that in subjects with pregnancy morbidity, including obstetric APS, elevated levels of HMGB1/sRAGE may represent an alarm signal, indicating an increase of proinflammatory triggers. Further studies are needed to evaluate the role of HMGB1/sRAGE as a possible tool to evaluate the risk stratification of adverse pregnancy outcomes.

## 1. Introduction

Inflammatory processes are implicated in every step of fertility, including early pregnancy (implantation and decidualization) [1]. However, recent evidence revealed that inflammatory triggers can lead to adverse pregnancy outcomes, such as preterm birth [2].

Understanding the mechanisms by which inflammation is untimely triggered in the uterus is fundamental to developing effective therapeutics to improve fertility and decrease poor obstetrical outcomes.

Recent studies have highlighted a close association between *high mobility group box 1 (*HMGB1), chronic inflammation, and autoimmune diseases [3, 4]. HMGB1 is a 30 kDa nuclear protein which organizes DNA and regulates transcription; it has been shown to play an important role in helping the recombination activating gene (RAG) endonuclease to form a complex during VDJ recombination. In addition, it was also found in cytosol, mitochondria, and cell plasma membrane, where it can be released to the extracellular milieu [5, 6]. In particular, during inflammation, HMGB1 may be secreted by immune cells, such as macrophages, monocytes, and dendritic cells. This molecule shows all the typical features of alarmins and plays a pivotal role in inducing and enhancing immune cell function, as well as in tissue injury and repair [7, 8]. HMGB1 can interact with Toll-like receptors (TLRs) and activates cells through multiple surface receptors, including Toll-like receptor 2 (TLR2), Toll-like receptor 4 (TLR4), the receptor for advanced glycation end-products (RAGE), leading to an upregulation of nuclear factor kappa-light-chain-enhancer of activated B cells (NF-kB), with production and release of cytokines, stimulating reactive oxygen species (ROS) release [9–12].

Recently, two isoforms of HMGB1 (28 and 30 kDa) have been characterized in human placenta and were shown to be highly expressed in preeclampsia [13]. Further studies revealed that the mRNA levels of HMGB1, RAGE, and NF-kB were increased in severe preeclamptic placentas. Moreover, increased level of HMGB1 was detected in sera of patients suffering from severe preeclampsia [14]. Other alarmins, including S100 calcium-binding protein A8 (S100A8), were also found to be elevated in early pregnancy loss [15]. However, the specific contribution of alarmins, mainly HMGB1, in these conditions is still under debate. Circulating increased HMGB1 levels have been shown during severe sepsis [16], pneumonia [17], systemic lupus erythematosus (SLE) [18–20], and in the synovial fluid of patients with rheumatoid arthritis [21]. In particular, in SLE patients, serum HMGB1 levels correlated with systemic lupus erythematosus disease activity index (SLEDAI), proteinuria, and anti-ds-DNA antibodies, showing a negative correlation with complement C3 [19, 22].

On the other hand, several autoimmune phenomena have been reported in a wide spectrum of obstetric complications, ranging from eclampsia to recurrent miscarriages [23]. In particular, in the pathogenesis of recurrent spontaneous abortion (RSA), immunological factors have been involved, such as decidual cells, complement system, cytokines, and genes of the histocompatibility complex that can determine the success or the failure of a pregnancy [24, 25]. A deeper insight into apparently unexplained recurrent spontaneous abortion shows increasing evidences supporting autoimmune mechanisms. The best-characterised pathogenic autoantibodies are antiphospholipid antibodies (aPL), and also other autoantibodies, such as anti-Ro/SSA and anti-La/SSB, have been found to be associated with an increased rate of abortion, poor pregnancy outcome, and several other obstetric manifestations [26, 27]. However, it is possible to find patients with RSA who persistently test negative for autoantibodies, including aPL [28–30].

Thus, in this investigation, we decided to analyze serum levels of HMGB1, which may be considered an endogenous sterile driver of inflammation and/or autoimmune response, in order to verify whether high levels of this molecule may represent an alarm signal for pregnancy morbidity.

## 2. Materials and Methods

### 2.1. Patients

The study included 30 consecutive patients, attending the Lupus Clinic, Rheumatology Unit of the Sapienza University of Rome, diagnosed as affected by APS according to the Sydney Classification Criteria [31]; they included both primary APS (*N* = 11) and APS associated with SLE (*N* = 19).

In addition, we enrolled as control group, 35 subjects affected by pregnancy morbidity tested persistently negative (at least 2 times 12 weeks apart) for conventional anticardiolipin (aCL) antibodies, anti-*β*_2_-glycoprotein I (a*β*_2_-GPI) antibodies, and lupus anticoagulant (LA) tests [29, 31].

Finally, 30 healthy women of fertile age (normal blood donors) were studied as controls. This study was approved by the local ethic committees and participants gave written informed consent. Sera were collected at several times and stored at −20°C until use.

### 2.2. ELISA for aCL and a*β*_2_-GPI Antibodies

aCL and a*β*_2_-GPI antibodies were tested in all the patients' and healthy donors' sera by enzyme-linked immunosorbent assay (ELISA) kits obtained from Inova Diagnostic Inc. (San Diego, CA, USA). ELISA was performed according to the manufacturer's instructions.

### 2.3. LA Assay

LA was studied in two coagulation systems, a dilute sensitized activated partial thromboplastin time (aPTT) and a dilute Russell's viper venom time (dRVVT), followed by a confirmation test using reagents and instrumentation by Hemoliance Instrumentation Laboratory, Lexington, MA, USA.

### 2.4. Western Blot

Sera (3 *μ*l) from subjects with pregnancy morbidity, patients with APS, and healthy donors were diluted with 72 *μ*l radioimmunoprecipitation assay (RIPA) buffer and heated at 95°C for 5 min in sodium dodecyl sulphate- (SDS-) loading buffer [32]. For immunodetection, the proteins were separated by 12.5% SDS-polyacrylamide gel electrophoresis (SDS-PAGE) and transferred onto polyvinylidene fluoride (PVDF) transfer membranes (Amersham Biosciences, Piscataway, NJ, USA). The membrane was blocked at room temperature for 1 h with Tris-buffered saline that contains 25 mM Tris-HCl, 150 mM NaCl, pH 7.4, and 0.05% Tween-20 (TBS-T) with 3% bovine serum albumin **(**BSA). The membranes were incubated with primary antibodies: anti-HMGB1 polyclonal antibody (1 : 1000; Abcam, Cambridge UK) or anti-RAGE monoclonal antibody (1 : 1000; Millipore, Billerica, MA, USA). The primary antibody was applied for 2 h at room temperature, followed by four 15 min washes with TBS-T. The secondary antibody was horseradish peroxidase-conjugated anti-rabbit (1 : 10,000; Sigma-Aldrich, Milan, Italy) or anti-mouse (1 : 5000; Amersham Biosciences) IgG, which was incubated for 1 h at room temperature. After washing, proteins were detected using ECL reagents (Amersham Biosciences). A standard sample was prepared by adding SDS buffer to human Jurkat cells and was included in each blot as an internal control. Densitometric analysis was performed using ImageJ software (National Institutes of Health, Bethesda, MD, USA).

### 2.5. Determination of Serum TNF-*α* Levels

Human tumour necrosis factor alpha (TNF-*α*) was tested in all the patients' and healthy donors' sera by the ELISA, using QuantiGlo Human TNF-*α* kit (R&D Systems Inc., Minneapolis, MN, USA). The minimal detectable level was 0.35 pg/mL.

### 2.6. Statistical Analysis

All the statistical procedures were performed by GraphPad Prism Software Inc. (San Diego, CA, USA). Normally distributed variables were summarized using the mean ± standard deviation (SD), and nonnormally distributed variables were by the median and range. Differences between numerical variables were tested with the Wilcoxon test. *p* values less than 0.05 were considered significant. Pearson's correlation coefficient (*r*) was used to assess correlation between sRAGE levels and HMGB1 levels.

## 3. Results

### 3.1. Characteristics of Patients

All 30 APS patients enrolled in this study were Caucasian females with a mean age of 34.3 years (range 17–49), and a mean disease duration of 5.5 years (range 0.1–16). The clinical characteristics of APS patients are reported in Table 1.

Subjects with pregnancy morbidity (*N* = 35) showed a mean age of 36.7 years (range 28–43); none of these subjects experienced thrombotic events. Among these subjects, 11 (27.5%) experienced fetal deaths, 1 (2.86%) premature births, and 25 (62.5%) three or more spontaneous abortions. In this group, two subjects had both spontaneous abortion and normal fetus deaths.

None of the healthy women of fertile age experienced arterial or venous thrombosis or pregnancy morbidity.

### 3.2. Analysis of Circulating HMGB1 in APS Patients and Subjects with Pregnancy Morbidity

**Figure 1:**
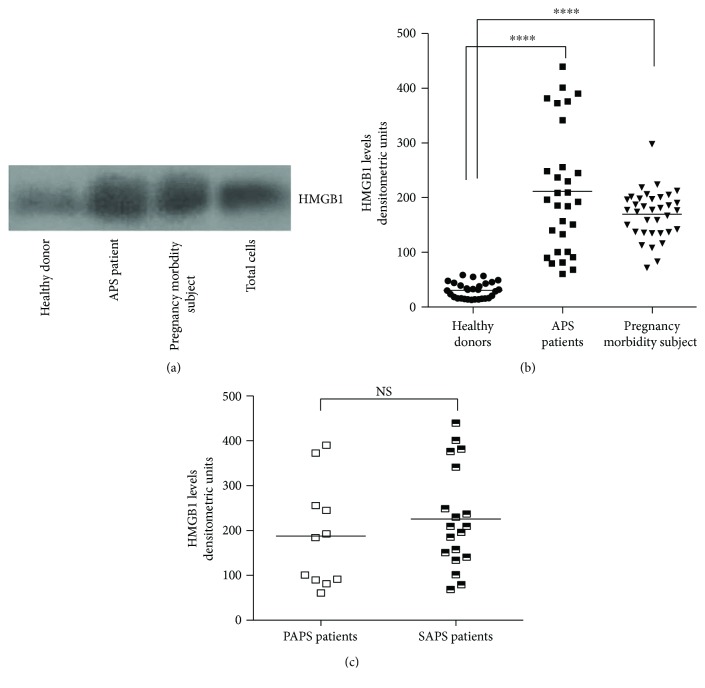
(a) Western blot analysis of HMGB1 expression in the serum of APS patients, subjects with pregnancy morbidity, and healthy donors. A lysate of Jurkat T cells (total cells) was analysed as a positive control. A representative blot for each group is shown. (b) Scatter plot analysis of HMGB1 expression levels in APS patients (*n* = 30), subjects with pregnancy morbidity (*n* = 35), and in healthy donors (*n* = 30). The data are presented as densitometric units. The horizontal bars indicate the mean. Serum HMGB1 levels from both APS patients and subjects with pregnancy morbidity were compared to healthy donors. ^∗∗∗∗^*p* < 0.0001. (c) Scatter plot analysis of HMGB1 expression levels in primary APS (PAPS) (*n* = 11) and secondary APS (SAPS) patients (*n* = 19). NS: not significant.

Since HMGB1 is an alarmin, whose circulating levels may be elevated during chronic inflammation, autoimmune diseases, or preeclampsia, in this investigation, we preliminarily tested HMGB1 expression by Western blot in sera from patients with APS patients, compared with women with pregnancy morbidity and healthy blood donors (Figure 1(a)). The results showed that virtually all the APS patients, either primary (PAPS) or secondary (SAPS), as well as the subjects with pregnancy morbidity showed increased serum levels of HMGB1, as compared to healthy women, as revealed by densitometric analysis (Figure 1(b)). Thus, HMGB1 serum levels of both APS patients and pregnancy morbidity subjects were significantly higher than healthy controls (*p* < 0.0001). Furthermore, no significant differences of HMGB1 levels between primary and secondary APS were found (Figure 1(c)).

Among APS patients, HMGB1 serum levels were not different in subjects with thrombotic events and in those with pregnancy morbidity; both of them presented serum HMGB1 levels significantly increased in comparison to healthy controls (*p* < 0.0001).

### 3.3. Analysis of sRAGE Levels in APS Patients and Subjects with Pregnancy Morbidity

**Figure 2:**
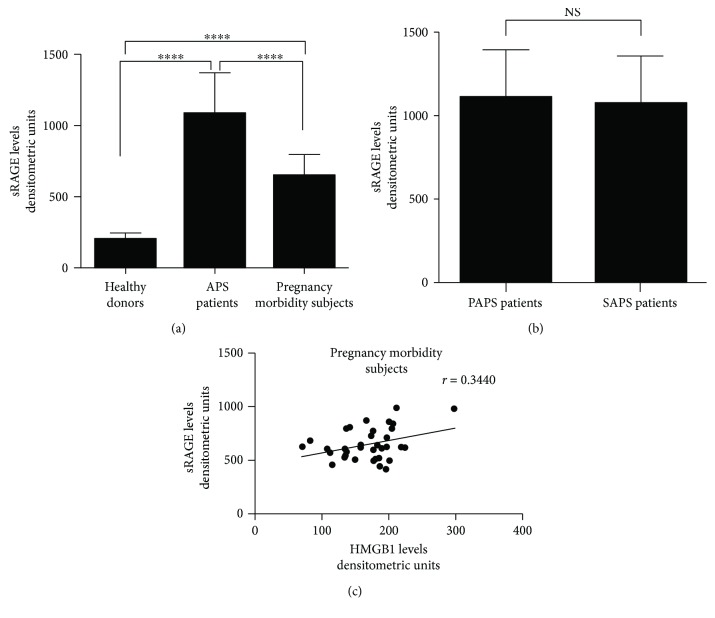
(a) Densitometric analysis of sRAGE levels detected by Western blot in APS patients (*n* = 30), subjects with pregnancy morbidity (*n* = 35), and healthy donors (*n* = 30). Bars represent the mean values; error bars indicate SD. ^∗∗∗∗^*p* < 0.0001. (b) sRAGE levels were detected by Western blot in PAPS (*n* = 11) and SAPS patients (*n* = 19). NS: not significant. (c) Scatter plot analysis of serum sRAGE levels versus serum HMGB1 levels in subjects with pregnancy morbidity. Statistically significant correlation was found between sRAGE and serum HMGB1 levels (*r* = 0.3440, *p* = 0.0430).

Since RAGE has been identified as the specific receptor for extracellular HMGB1, we further analyzed soluble RAGE (sRAGE) in sera of APS patients, subjects with pregnancy morbidity, and healthy blood donors. The results showed that both APS patients and subjects with pregnancy morbidity showed significantly increased levels of sRAGE as compared to healthy women (*p* < 0.0001, Figure 2(a)). No significant differences of serum sRAGE levels between primary and secondary APS were detected (Figure 2(b)).

A significant correlation was found between HMGB1 and sRAGE levels in the subjects with pregnancy morbidity (*r* = 0.3440, Figure 2(c)).

### 3.4. Analysis of TNF-*α* Levels in APS Patients and Subjects with Pregnancy Morbidity

**Figure 3:**
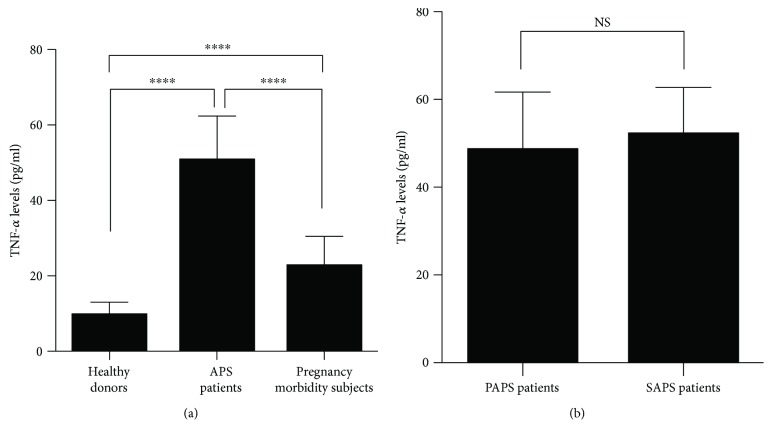
(a) Detection of serum TNF-*α* levels by ELISA. Serum TNF-*α* levels from APS patients, subjects with pregnancy morbidity, and healthy donors were compared. ^∗∗∗∗^*p* < 0.0001. (b) Serum TNF-*α* levels were analyzed in PAPS (*n* = 11) and SAPS patients (*n* = 19) by ELISA. NS: not significant.

We then decided to test TNF-*α* levels, considering this molecule as a possible proinflammatory cytokine in APS patients. The highest values were detected in sera of APS patients. Statistical analysis revealed that TNF-*α* levels of APS patients were significantly higher as compared to both pregnancy morbidity subjects and healthy donors (*p* < 0.0001, Figure 3). However, also the pregnancy morbidity subjects showed significantly increased levels of TNF-*α* as compared to healthy women (*p* < 0.0001) (Figure 3(a)). We did not find significant differences in TNF-*α* levels between primary and secondary APS (Figure 3(b)).

## 4. Discussion

In this study, we show elevated serum levels of the alarmin HMGB1 in patients with APS and in subjects with pregnancy morbidity. As a consequence, we also found in both groups significantly increased levels of sRAGE, the putative receptor for extracellular HMGB1.

These findings are not surprising, since it is well known that HMGB1 may play a role in inducing and enhancing innate immunity, and, secondly, inflammatory and autoimmune phenomena may be involved in a wide spectrum of obstetric complications [7, 13, 14, 33].

In particular, we show for the first time the increased levels of HMGB1 in APS patients. Interestingly, we observed this phenomenon not only in secondary APS (elevated levels of HMGB1 during SLE have already been reported) [19, 34] but also in primary APS. During pregnancy, proinflammatory stimuli have been associated with higher risk of adverse pregnancy outcomes, such as preterm birth [2, 35]. In particular, HMGB1, once accumulated in the extracellular milieu, is able to convey danger signals by triggering inflammatory patterns with extracellular signal-regulated kinases (ERKs), p38, and NF-kB activation via several cell surface receptors, including TLR2, TLR4, CD24, and, mainly, RAGE [12, 16, 36].

From a technical point of view, we decided to test HMGB1 by Western blot, instead of ELISA, to avoid the possibility that serum/plasma components able to bind to HMGB1 may interfere with its detection [32].

To confirm that also in our patients HMGB1/RAGE system represents an endogenous “driver” of inflammation, we tested the levels of TNF-*α*, the most typical proinflammatory cytokine, and observed that APS patients showed significantly increased levels of TNF-*α*, as compared to control subjects. Thus, we can conclude that also in APS patients the increase of serum level of HMGB1 is accompanied by high levels of TNF-*α*. It is in agreement with similar observation in SLE patients, where elevated plasma level of HMGB1 is associated with disease activity and combined alterations with TNF-*α* [20].

As several studies have shown elevated levels of HMGB1 in pregnancies at high risk of developing complications associated with placental dysfunction such as growth restriction or preterm labor [37] and preeclampsia [38] for the first time, we also aimed to evaluate serum levels of this alarmin in subjects with pregnancy morbidity.

Our findings suggest that in subjects with pregnancy morbidity, including obstetric APS, elevated levels of HMGB1/sRAGE may represent an alarm signal, indicating an increase of proinflammatory triggers. However, this finding cannot be considered highly specific, since we ourselves reported that several events, including surgical/anesthesia trauma, induce an early intracellular upregulation of HMGB1 in monocytes, with consequent release of the alarmin in serum [39].

In conclusion, HMGB1/sRAGE may play a role in monitoring of pregnancy morbidity risk. Additional studies are needed to demonstrate that monitoring HMGB1/sRAGE together with other prognostic parameters may represent a useful tool to evaluate the risk stratification, in order to prevent adverse pregnancy outcomes.

## Figures and Tables

**Table 1:** Clinical characteristics of APS patients.

Characteristics, *n* (%)	APS (*n* = 30)	SAPS (*n* = 19)	PAPS (*n* = 11)
Vascular thrombosis	28 (93.3)	19 (100)	9 (81.8)
Venous thrombosis	18 (60)	13 (68.4)	5 (45.4)
Arterial thrombosis	13 (43.3)	8 (42.1)	5 (45.4)
Recurrent thrombosis	12 (40)	9 (47.4)	3 (27.3)

Pregnancy morbidity	9 (30)	5 (26.3)	4 (36.4)
Normal fetus deaths	2 (6.7)	1 (5.26)	1 (9.1)
Premature births	0	0	0
Spontaneous abortions	8 (26.7)	5 (26.3)	3 (27.3)

Vascular thrombosis and pregnancy morbidity	7 (23.3)	5(26.3)	2 (18.2)

Noncriteria APS features	24 (80)	16 (84.2)	24 (80)
Livedo reticularis	12 (40)	8 (42.1)	4 (36.4)
Thrombocytopenia	7 (23.3)	4 (21)	3 (27.3)
Migraine	7 (23.3)	6 (31.6)	1 (9.1)
Seizures	4 (13.3)	3 (15.8)	1 (9.1)

APS: antiphospholipid syndrome; PAPS: primary APS; SAPS: secondary APS.
